# Sumac (*Rhus coriaria L.*) Supplementation on High-Sensitivity C-Reactive Protein Concentrations in Adults: A Systematic Review and Dose-Response Meta-Analysis

**DOI:** 10.1016/j.cdnut.2025.107555

**Published:** 2025-09-07

**Authors:** Mostafa Shahraki Jazinaki, Hossein Bahari, Iman Rahnama, Mohammad Safarian, Mahsa Malekahmadi

**Affiliations:** 1 Department of Nutrition, Faculty of Medicine, Mashhad University of Medical Sciences, Mashhad, Iran; 2 Student Research Committee, Mashhad University of Medical Sciences, Mashhad, Iran; 3 Imam Khomeini Hospital Complex, Tehran University of Medical Sciences, Tehran, Iran

**Keywords:** sumac, high-sensitivity C-reactive protein, inflammation, systematic review, meta-analysis

## Abstract

**Background:**

Chronic low-grade inflammation is implicated in the development of various metabolic and cardiovascular disorders. Sumac (*Rhus coriaria* L.) is a medicinal plant with potential anti-inflammatory properties. However, the effects of sumac supplementation on inflammatory biomarkers, particularly high-sensitivity C-reactive protein (hs-CRP), remain controversial.

**Objectives:**

This systematic review and meta-analysis aimed to evaluate the effect of sumac supplementation on hs-CRP concentrations in adults.

**Methods:**

A systematic search was performed in PubMed, Scopus, Web of Science, and the Cochrane Library databases up to February 2025 to identify RCTs examining the impact of sumac supplementation on hs-CRP concentrations. Screening, data extraction, and risk of bias assessment were conducted independently. A random-effects model was used to calculate the overall effect size as the SMD with 95% CIs.

**Results:**

Seven RCTs were eligible for inclusion in this review. The pooled analysis demonstrated a significant reduction in hs-CRP concentrations following sumac supplementation compared with that in the control groups (SMD: −0.33; 95% CI: −0.64, −0.02; *P* = 0.03). Moreover, a significant heterogeneity was detected among the pooled effect sizes (*P* = 0.02; *I*^2^ = 60.2%). Subgroup analyses revealed that sumac supplementation led to a significant reduction in hs-CRP concentrations in trials that included both genders or in studies conducted on patients with nonalcoholic fatty liver disease, individuals with overweight, or those aged ≥45 y.

**Conclusions:**

Sumac supplementation may significantly reduce hs-CRP concentrations in adults. Further high-quality, large-scale trials in various populations in terms of areas or health conditions are required to confirm these findings and determine the optimal dosage and duration for sumac supplementation to reduce inflammation.

This trial is registered at PROSPERO database as CRD420251016622.

## Introduction

Inflammation is defined as a physiological response of the immune system triggered by harmful stimuli, including damaged cells, pathogens, irradiation, or toxic compounds, which is vital to maintain health [1,2]. The role of systemic low-grade inflammation was identified in the pathogenesis of chronic noncommunicable diseases, such as type 2 diabetes mellitus (T2DM), specific cancer types, and cardiovascular diseases (CVDs) [3]. This highlights the critical involvement of inflammation in the progression of such diseases [4], underscoring the need for further research and targeted interventions to mitigate its impact [5]. To assess the level of inflammation in the body, various biomarkers are commonly measured, including TNF-α, C-reactive protein (CRP), and IL-6 [6]. Previous studies reported that elevated concentrations of inflammatory markers, primarily TNFα, CRP, and IL-6, are often associated with the progression of diseases such as CVDs, cancer, and T2DM [[7], [8], [9], [10]].

High-sensitivity C-reactive protein (hs-CRP) is recognized as a biomarker for assessing inflammation that could be associated with the development of various health conditions [11]. In addition, hs-CRP is identified as a predictive marker of stroke and cardiovascular events [12,13] and is commonly used in clinical practice to detect low-grade inflammation [14]. According to available evidence, dietary components could lead to the improvement or exacerbation of inflammation in the body [15]. In recent years, studies on medicinal plants and their phytochemicals have resulted in finding new agents with anti-inflammatory properties [15].

Sumac (*Rhus coriaria L.*) is a plant from the Anacardiaceae family that is native to the Mediterranean and widely distributed. According to folklore medicine, although sumac fruit is consumed to treat ulcers, urinary system issues, diarrhea, and liver disease, sumac powder is usually prescribed to reduce cholesterol concentrations [16]. However, it is commonly used as a spice [17]. Thus far, >200 phytochemicals have been extracted from *R coriaria*, such as anthocyanins [18], isoflavonoids [19], flavonoids [20], and phenolic acids [16,21]. The anti-inflammatory properties of these components are reported in recent studies. Such findings highlight the potential of sumac as a natural therapeutic agent in managing inflammation.

Although several RCTs have reported promising results regarding the effects of sumac consumption on reducing hs-CRP concentrations, some studies have presented inconsistent findings. To address this gap and provide a comprehensive understanding, our study aimed to conduct a systematic review and meta-analysis to evaluate the impacts of sumac supplementation on hs-CRP concentrations.

## Methods

All steps of conducting this systematic review and meta-analysis were performed based on the PRISMA framework [22]. Moreover, the protocol of this review is registered in the PROSPERO database (CRD420251016622).

### Search strategy

Web of Science ISI, PubMed, Scopus, and Cochrane Library databases were comprehensively searched up to February 2025 to find relevant RCTs that investigated the impacts of sumac supplementation on hs-CRP concentrations. The search strategy used in this search included the following terms: (“sumac”) OR (“Rhus”) AND (“clinical trial”) OR (“randomized”) OR (“randomized controlled trial”) OR (“RCT”) OR (“placebo”). Details of the performed search strategy in each database are provided in Supplemental Table 1. No date or language restrictions were applied to our search. All found papers were accurately screened using the EndNote software based on their titles and abstracts by 2 authors (HB and MSJ), independently. The screening step was performed according to PICOS (participant: adults; intervention: sumac supplementation; comparison: control group; outcome: hs-CRP concentrations; study type: RCTs) [23]. We discussed any controversies until we reached a consensus.

### Eligibility criteria

All included trials met the following criteria: *1*) RCTs assessing impacts of Sumac intake on hs-CRP concentrations (without combined treatments); *2*) there was an appropriate control group; *3*) the intervention should have been performed on adults (≥18 y); *3*) the mean change and SD of the changes in hs-CRP concentrations should be reported (or be possible to calculate using formulas).

### Exclusion criteria

Trials with combined treatment along with sumac supplementation, conducting intervention on nonadults (<18 y), or without a suitable control group; animal studies; observational research including crossover, case control, and cohort studies; protocol articles, review articles; short communications; or letters to editors were excluded from this review. To prevent missing related trials, references from all eligible studies were checked. In addition, the Google Scholar search engine was manually searched.

### Data extraction

The relevant data for the topic of this research were extracted from eligible articles by 2 authors (MSJ and HB), independently, followed by the screening process. Data included the first author’s name, date of publication, the country that was the origin of this research, total sample size and the number of participants in each group, features of populations [health status, mean age, and mean BMI (in kg/m^2^)], characteristics of intervention (type, dosage, and duration of sumac supplementation), type of the control group, and mean change and SD of changes in hs-CRP concentrations followed by sumac supplementation or in the control group. Any disagreement or conflict was discussed until a consensus was reached.

### Risk of bias assessment

Risk of bias assessment was performed based on the Revised Cochrane risk of bias tool for randomized trials (RoB 2.0) by 2 authors (MSJ and HB), independently [24,25]. This framework assessed risk of bias in the following 5 domains: bias arising from the randomization process, bias due to deviations from intended interventions, bias due to missing outcome data, bias in measurement of the outcome, and bias in selection of the reported result. Risk of bias levels in the domain could be considered as low, some concerns, and high. Disagreements were resolved in consultation with the third author (MM).

### Data synthesis and statistical analysis

All analyses in this research were performed by Stata, version 17.0 (Stata Corp). Moreover, in all analyses, *P* values of <0.05 were considered statistically significant. Overall effect size in this meta-analysis was calculated according to the random-effects model by using the mean changes and SDs of hs-CRP concentration changes in the intervention and control groups. Moreover, the pooled effect size was expressed as a SMD with a 95% CI. In the cases where mean changes were not reported directly, mean changes were calculated by subtracting the baseline from the final values. Further, when the SD of mean changes was not reported, it was estimated by using the following formula: SD = square root [(SD_baseline_)^2^ + (SD_final_)^2^ − (2 × R × SD_baseline_ × SD_final_)] [26]. If 95% CIs, IQRs, or SEs were reported instead of SDs, SDs were calculated on the basis of the method by Hozo et al. [27]. Heterogeneity among the included trials was investigated by carrying out Cochran Q test and measures by interpretation of *I*^2^ statistics [28]. A *P* value of <0.05 and *I*^2^ of >50% were identified as significant heterogeneity. Subgroup analysis performed to find the sources of heterogeneity was based on the following predefined criteria [29]: intervention duration (<12 and ≥12 wk), sumac supplementation dosage (<3 and ≥3 g/d), health status [T2DM, nonalcoholic fatty liver disease (NAFLD), and others], genders (both sexes and females alone), age (<45 and ≥45 y), and baseline BMI (overweight and obesity). Sensitivity analysis was performed to evaluate the impact of omitting each of the included trials on the overall effect size on the basis of the leave-1-out method [30]. The possible publication bias was assessed by performing Egger regression and visual interpretation of funnel plots [31]. The linear relationship between the features of sumac supplementation (dosage and duration) and changes in the hs-CRP concentrations was evaluated by performing the meta-regression analysis [32]. Furthermore, by conducting the dose-response analysis (fractional polynomial modeling), the nonlinear relationship between dosage or duration of sumac supplementation and hs-CRP concentration changes was evaluated [33].

### Certainty assessment

The quality assessment of the evidence was performed based on the Grading of Recommendations Assessment, Development, and Evaluation protocol [34]. On the basis of this framework, the quality of the evidence was evaluated in 5 domains—imprecision, risk of bias, inconsistency, publication bias, and indirectness. The levels of limitations in each domain were categorized into the following 3 levels: no serious, serious, and very serious. Finally, the overall evidence quality was rated into the following 4 grades: very low, low, moderate, and high.

## Results

### Study selection

**FIGURE 1:**
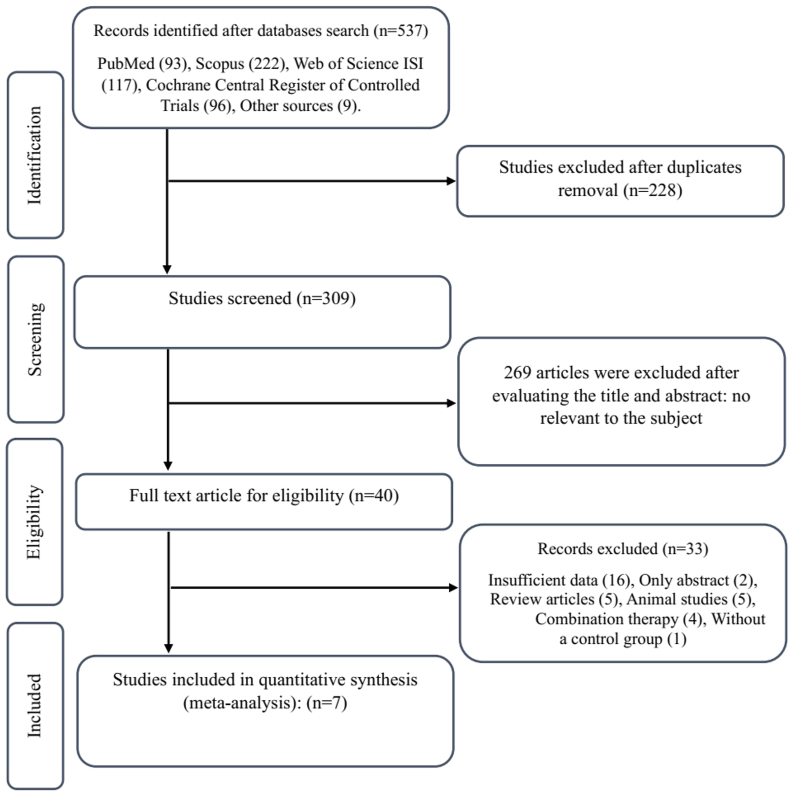
Flowchart of study selection for inclusion trials in the meta-analysis.

Of 537 articles identified through a comprehensive search in databases, 228 duplicated items were removed. Then, 309 remaining trials were screened based on their titles and abstracts, leading to the exclusion of 269 trials that did not meet our review inclusion criteria. Reading the full texts was mandatory for deciding on 40 studies, leading to the exclusion of 33 articles due to being only abstracts (n = 2), insufficient data (n = 16), review articles (n = 5), animal studies (n = 5), combination treatments (n = 4), and without a control group (n = 1). Finally, 7 studies were eligible for inclusion in this systematic review and meta-analysis [[35], [36], [37], [38], [39], [40], [41]]. The flowchart of study selection is presented in Figure 1.

### Study characteristics

**FIGURE 2:**
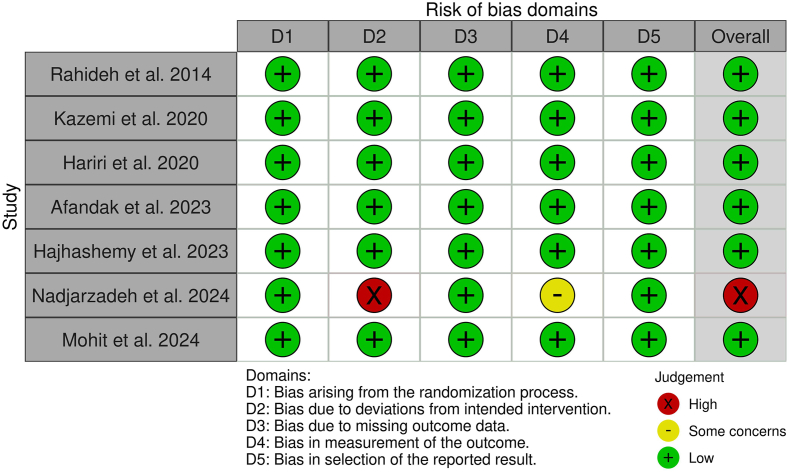
Risk of bias plot for included trials.

In total, 7 trials were eligible for inclusion in this review [[35], [36], [37], [38], [39], [40], [41]]. Included studies were published between 2014 [35] and 2024 [40,41]. All included studies were conducted in Iran. The design of a trial conducted by Hajhashemy et al. [39] was crossover, whereas the rest of the studies had a parallel design. The sample sizes of eligible studies were varied from 32 [39] to 80 individuals [37]. The mean age and baseline BMI of participants ranged from 24.5 [38] to 58.7 y [39], and 25.6 [38] to 31.6, respectively. The population of trials included individuals with T2DM [35,41], NAFLD [37,40], polycystic ovary syndrome [38], metabolic syndrome [39], and overweight or obese females with depression [36]. The duration and dosage of sumac powder supplementation were in the range of 6 [39] to 12 wk [[35], [36], [37], [38],^41^] and 1 [39] to 6 g/d [41]. The characteristics of the included trials are summarized in Table 1 [[35], [36], [37], [38], [39], [40], [41]]. Risk of bias assessment that was performed based on the RoB 2 guideline identified the overall risk of bias as high for 1 trial [41], whereas the rest of them had a low overall risk of bias. In the study conducted by Nadjarzadeh et al. [41], levels of risk of bias in domains of deviations from intended interventions and measurement of outcomes were high and some concerns, respectively. However, risk of bias in other domains for this trial was identified as low. Further, remaining eligible studies had a low risk of bias in all RoB 2 domains. The details of the risk of bias assessment in each subclass are exhibited in the plot of risk of bias assessment (Figure 2).

**TABLE 1:** Characteristic of included studies in meta-analysis.

Studies	Country	Study design	Participant	Samplesize andgender	Samplesize		Trial duration (wk)	Age (y), mean ± SD		BMI (kg/m^2^), mean ± SD		Intervention	
					IG	CG		IG	CG	IG	CG	Sumac powder dose (g/d)	Control group
Rahideh et al. [35], 2014	Iran	Parallel, R, PC, DB	Type 2 diabetes mellitus	41; B	22	19	12	46.1 ± 8.5	47.5±8.4	29.5 ± 2.8	29.5 ± 2.2	Sumac powder; 3 g/d	Placebo
Kazemi et al. [37], 2020	Iran	Parallel, R, PC, DB	Nonalcoholic fatty liver disease	80; B	40	40	12	41.8 ± 10.6	41.4 ± 9.7	27.6 ± 1.4	27.5 ± 1.6	Sumac powder 2 g/d + 500 kcal calorie-deficient	Placebo + 500 kcal calorie-deficient
Hariri et al. [36], 2020	Iran	Parallel, R, PC, DB	Overweight or obese females with depression	62; F	31	31	12	42.1 ± 8.4	44.0 ± 11.8	32.4 ± 3.7	31.2 ± 3.8	Sumac powder 3 g/d + restricted calorie diet	Placebo + restricted calorie diet
Afandak et al. [38], 2023	Iran	Parallel, R, PC, DB	Polycystic ovary syndrome	75; F	39	36	12	23.0^1^ ± 5.1	26.0^1^ ± 4.4	25.7^1^ ± 2.6	25.6^1^ ± 2.4	Sumac powder; 3 g/d	Placebo
Hajhashemy et al. [39], 2023	Iran	Cross over, R, PC, TB	Metabolic syndrome	32; B	32	32	6	58.7 ± 5.8	58.7 ± 5.8	31.6 ± 4.6	31.6 ± 4.6	Sumac powder; 1 g/d	Placebo
Nadjarzadeh et al. [41], 2024	Iran	Parallel, R, C, SB	Type 2 diabetes mellitus	58; B	30	28	12	52.3 ± 7.05	51.6 ± 7.07	29.1 ± 5.3	28.6 ± 4.1	Sumac powder + low-fat yogurt; 6 g/d	Low-fat yogurt
Mohit et al. [40], 2024	Iran	Parallel, R, PC, DB	Nonalcoholic fatty liver disease	55; B	23	22	8	45.0^1^ ± 11.8	46.0^1^ ± 15.0	29.4^1^ ± 2.5	29.0^1^ ± 2.4	Sumac powder; 3 g/d	Placebo

*Abbreviations:* B, both; CG, control group; CO, controlled; DB, double-blinded; F, Female; IG, intervention group; PC, placebo-controlled; R, randomized; SB, single-blinded; TB, triple-blind; M, Male.

1 Median.

### Meta-analysis

#### Effect of sumac supplementation on hs-CRP concentrations

**FIGURE 3:**
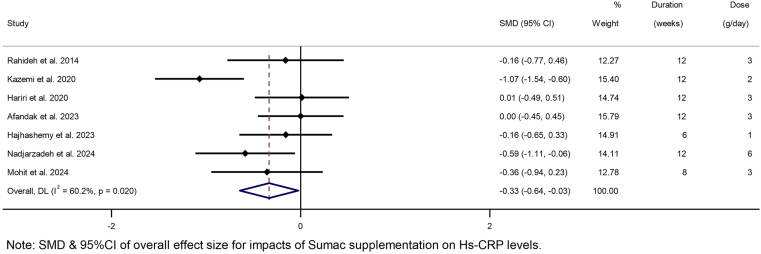
Forest plot detailing SMD and 95% CIs for the effect of sumac intake on high-sensitivity C-reactive protein (hs-CRP).

Pooling 7 effect sizes revealed sumac powder supplementation led to a significant decrease in hs-CRP concentrations compared with the control treatment (SMD: −0.33; 95% CI: −0.64, −0.02; *P* =0.03) (Figure 3). However, there was a significant heterogeneity among combined effect sizes (*P* = 0.02; *I*^2^ = 60.2%). The subgroup analysis showed a significant decrease in hs-CRP concentrations followed by sumac supplementation in individuals with NAFLD, overweight, or aged ≥ 45 y or in trials that included both sexes (Table 2).

**TABLE 2:** Subgroup analyses of Sumac supplementation on hs-CRP in adults.

Subgroup analyses of sumac supplementation on hs-CRP	*N*	SMD (95% CI)	*P*	Heterogeneity		
				*P*-heterogeneity	*I*^2^ (%)	*P* between subgroups
Overall effect	7	−0.33 (−0.64, −0.02)	0.03^1^	0.02^1^	60.2	
Age						
<45	3	−0.35 (−1.05, 0.35)	0.32	0.001^1^	85.0	0.92
≥45	4	−0.31 (−0.59, −0.04)	0.02^1^	0.64	0.0	
Trial duration (wk)						
<12	2	−0.23 (−0.61, 0.13)	0.21	0.61	0.0	0.66
≥12	5	−0.36 (−0.79, 0.06)	0.09	0.006^1^	72.3	
Intervention dose (g/d)						
<3	2	−0.61 (−1.51, 0.27)	0.17	0.008^1^	85.6	0.37
≥3	5	−0.19 (−0.43, 0.04)	0.10	0.43	0.0	
Health status						
T2DM	2	−0.40 (−0.81, 0.01)	0.05	0.30	6.3	0.11
NAFLD	2	−0.73 (−1.43, −0.03)	0.03^1^	0.06	71.0	
Others	3	−0.04 (−0.32, 0.23)	0.74	0.86	0.0	
Gender						
Both sexes	5	−0.48 (−0.84, −0.12)	0.008^1^	0.06	55.7	0.05
Female	2	0.006 (−0.32, 0.34)	0.97	0.97	0.0	
Baseline BMI						
Overweight (25–29.9)	5	−0.44 (−0.84, −0.04)	0.03^1^	0.02^1^	65.8	0.17
Obese (>30)	2	−0.07 (−0.42, 0.27)	0.68	0.63	0.0	

*Abbreviations:* hs-CRP, high-sensitivity C-reactive protein; NAFLD, nonalcoholic fatty liver disease; T2DM, type 2 diabetes mellitus.

1 Statistically significant (*P* < 0.05).

#### Meta-regression and dose-response analysis

**FIGURE 4:**
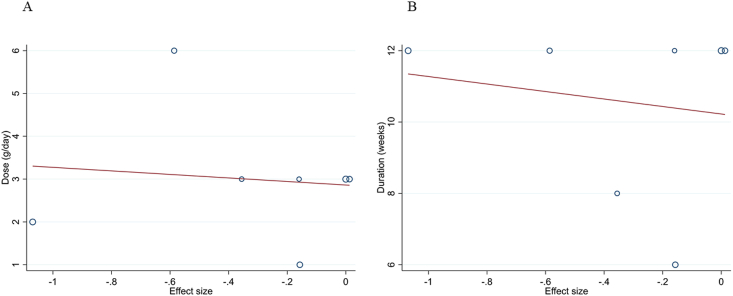
Random-effects meta-regression plots of the association between mean changes in high-sensitivity C-reactive protein concentrations and (A) sumac dose (g/d) and (B) intervention duration (weeks).

**FIGURE 5:**
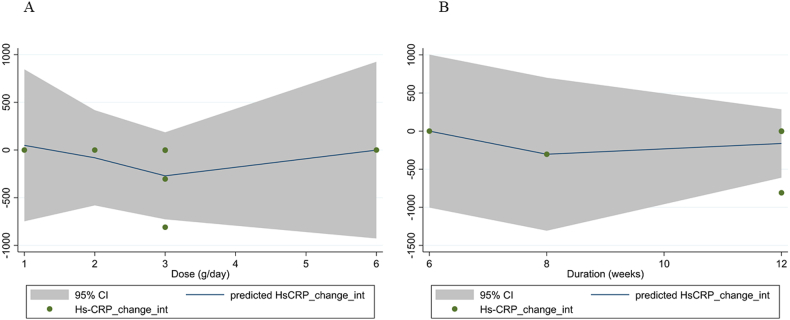
Dose-response relations between (A) sumac dosage (g/d) and (B) duration of sumac supplementation (weeks) and mean difference in high-sensitivity C-reactive protein (hs-CRP) concentrations.

Meta-regression showed no significant linear relationship between dosage and duration of sumac supplementation with changes in hs-CRP concentrations (*P*-linearity = 0.82 and *P*-linearity = 0.72, respectively) (Figure 4A, B). Moreover, fractional polynomial modeling (dose-response analysis) demonstrated no significant nonlinear relationship between features of sumac supplementation, including dosage and duration, and hs-CRP concentration changes (*P*-nonlinearity = 0.38 and *P*-nonlinearity = 0.62, respectively) (Figure 5A, B).

#### Sensitivity analysis and publication bias

**FIGURE 6:**
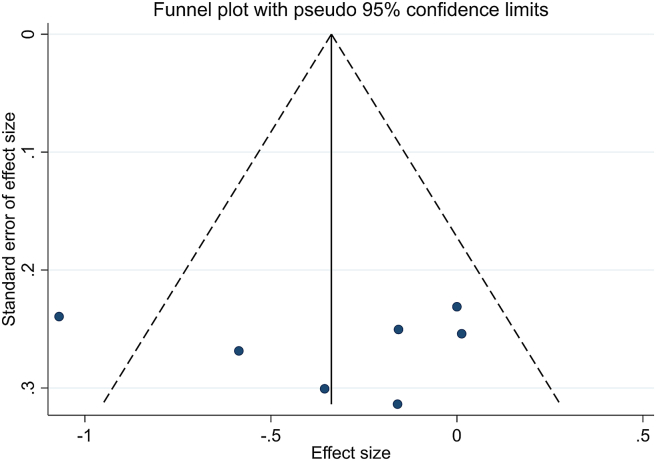
Funnel plots for the effect of sumac intake on high-sensitivity C-reactive protein concentrations.

Sensitivity analysis demonstrated a significant change in the overall effect size for impacts of sumac supplementation on hs-CRP after omitting the study by Kazemi et al. (SMD: −0.18; 95% CI: −0.39, 0.02) [37], Nadjarzadeh et al. (SMD: −0.29; 95% CI: −0.64, 0.05) [41], or Mohit et al. (SMD: −0.33; 95% CI: −0.68, 0.02) [40]. Egger regression (*P*-Egger = 0.87) and visually inspected funnel plot indicated no significant publication bias for studies investigating the impact of sumac supplementation on hs-CRP concentrations (Figure 6).

#### Certainty assessment

The evidence quality assessment that was performed according to the Grading of Recommendations Assessment, Development, and Evaluation protocol showed that the level of evidence was downgraded to moderate due to serious inconsistency. The details of evidence quality assessment in each domain are presented in Table 3.

**TABLE 3:** GRADE profile of sumac supplementation for hs-CRP.

Quality assessment						Quality of evidence
Outcomes	Risk of bias	Inconsistency	Indirectness	Imprecision	Publication bias	
hs-CRP	No serious limitations	Serious limitations^1^	No serious limitations	No serious limitations	No serious limitations	⊕⊕⊕◯ Moderate

*Abbreviations:* GRADE, Grading of Recommendations Assessment, Development, and Evaluation; hs-CRP, high-sensitivity C-reactive protein.

1 There was a moderate heterogeneity among the included studies (*I*^2^= 60.2).

## Discussion

Sumac (*R coriaria* L.) is a widely used spice in the Mediterranean region, known for its rich phytochemical content, including flavonoids, tannins, and phenolic acids, which contribute to its potential health benefits [16,42]. Epidemiologically, sumac is utilized in traditional medicine for managing various ailments such as hemorrhoids, wound healing, and inflammation. Its consumption has been associated with beneficial effects on metabolic syndrome, oxidative stress, and inflammation, which are key factors in conditions such as CVD, diabetes, and cancer [16,39,42]. Clinical trials have shown that sumac can reduce oxidative stress markers and improve antioxidant capacity, although its effects on inflammation are less consistent, with some studies reporting no significant changes in inflammatory markers like TNF-α and IL-6 [39,43]. However, sumac has demonstrated potential in reducing inflammation in specific conditions, such as gastritis and gouty arthritis, by modulating inflammatory pathways [17,44]. Overall, sumac’s rich phytochemical profile and its potential to manage oxidative stress and inflammation make it a promising candidate for further research in the context of metabolic and inflammatory diseases [16,42,43].

**FIGURE 7:**
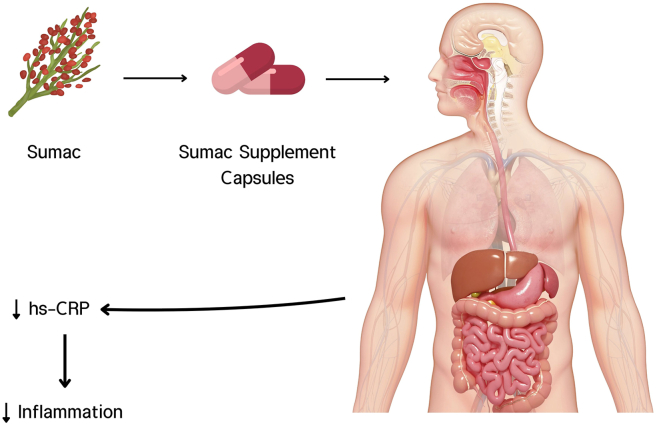
Sumac supplementation significantly reduced high-sensitivity C-reactive protein (hs-CRP) concentrations in adults.

In the current systematic review and meta-analysis, which examined 7 RCTs in Iran, it was shown that intervention with sumac significantly reduced hs-CRP concentrations in comparison with the control group (Figure 7). The subgroup analysis confirmed this effectiveness in trials that included both genders or in those that were conducted on individuals with NAFLD, overweight, or aged ≥ 45 y.

A systematic review of randomized clinical trials found inconsistent results regarding the impact of sumac on hs-CRP concentrations, with some studies reporting a decrease, whereas others finding no significant change [43]. In an RCT involving patients with NAFLD, sumac supplementation led to a significant reduction in hs-CRP concentrations, suggesting a potential anti-inflammatory effect [37]. Similarly, a study on females with polycystic ovary syndrome reported a significant decrease in hs-CRP concentrations following sumac supplementation [38]. Conversely, another trial in adults with metabolic syndrome found no significant effect of sumac on inflammation markers, including hs-CRP [39]. The differences in results could be attributed to variations in study populations, dosages, and durations of sumac supplementation, as well as differences in baseline inflammatory status among participants. These factors may influence the extent to which sumac can modulate inflammatory responses, highlighting the need for further research to clarify its effects on CRP and other inflammatory markers.

The potential mechanism of action involves its antioxidant properties, which help in managing oxidative stress and inflammation. The phytochemicals in sumac, such as tannins and flavonoids, are known to enhance antioxidant capacity, thereby reducing oxidative stress markers such as malondialdehyde and increasing total antioxidant capacity [39,43]. Additionally, certain studies have indicated that sumac exhibits antioxidant properties in cell-free models of oxidative stress by preventing lipid peroxidation and the generation of superoxide anions through the xanthine/xanthine oxidase system [45]. Earlier research has also reported that extract from *Rhus verniciflua* Stokes may mitigate Catalase (CAT) depletion in macrophage cells and lower thiobarbituric acid reactive substances and intracellular reactive oxygen species concentrations induced by H_2_O_2_ [46]. These effects collectively contribute to the anti-inflammatory action of sumac, potentially lowering hs-CRP concentrations and improving metabolic health.

### Strengths and limitations

To our knowledge, this is the first meta-analysis to investigate the effect of sumac on hs-CRP concentrations. In addition, assessing risk of bias of the included trials by using the RoB 2 tool, low general risk of bias in the majority of the included studies, certainty assessment of evidence according to the GRADE framework, conductive comprehensive subgroup analysis to find possible sources of heterogeneity, performing meta-regression, and dose-response analysis were other strengths of this review. However, this review had some limitations, including the small number of included trials, the high general risk of bias in the 1 included trial, limited sample sizes, and significant heterogeneity and inconsistency among the eligible studies. Further, the overall effect size changed significantly after omitting each of the 3 effect sizes of the studies by Kazemi et al. [37], Nadjarzadeh et al. [41], or Mohit et al. [40]. In addition, this review focused on the impacts of sumac supplementation on changes in hs-CRP concentrations, and assessing its influence on other inflammatory markers was not feasible due to the limited number of relevant RCTs. Furthermore, because all eligible studies were conducted in Iran, generalizing findings to non-Iranian populations may require caution. So, the study suggests conducting RCTs to assess the impacts of sumac supplementation on various inflammatory markers in diverse geographic populations to reach a firm conclusion regarding its effectiveness.

## Conclusion

This systematic review and meta-analysis revealed that sumac supplementation significantly reduces hs-CRP concentrations compared with control groups. However, owing to borderline significance levels, the limited number of included studies, and serious inconsistency in evidence, these findings should be interpreted cautiously. More well-designed RCTs with larger sample sizes are needed to draw definitive conclusions regarding the sumac’s anti-inflammatory effects.

## Author contributions

The authors’ responsibilities were as follows – MSJ conceived and designed the research; MSJ, HB: performed screening and data extraction; MSJ, MM, IR: drafted the manuscript; MS, HB, MM: revised the manuscript; and all authors: have read and agreed to the published version of the manuscript.

## Data availability

All data generated or analyzed during this study are included in this published article.

## Funding

The authors declare that no funds, grants, or other support were received during the preparation of this manuscript.

## Conflicts of interest

The authors report no conflicts of interest.
