# Supplementary material for: Sumac (Rhus coriaria L.) Supplementation on High-Sensitivity C-Reactive Protein Concentrations in Adults: A Systematic Review and Dose-Response Meta-Analysis
Source: Curr Dev Nutr. 2025 Sep 7;9(10):107555. doi: 10.1016/j.cdnut.2025.107555 (PMC12504994; doi:10.1016/j.cdnut.2025.107555)
Supplement: multimedia component 1 [file mmc1.docx]

**Sumac (*Rhus coriaria L.*) Supplementation on High-sensitivity C-reactive Protein (Hs-CRP) Levels in Adults: A Systematic Review and Dose-response Meta-analysis
Mostafa Shahraki Jazinaki, et. al**

Electronic Supplementary Material Appendix Tables S1

**Electronic Supplementary Material** **Appendix Table S1**. Search strategy to find potentially eligible trials for inclusion in a meta-analysis of Sumac supplementation and high-sensitivity C-reactive Protein (Hs-CRP).

| **Search strategy** |
| --- |
| **PubMed**: (((((((((((((randomized[Title/Abstract]) OR (placebo[Title/Abstract])) OR (clinical trials[Title/Abstract])) OR (randomly[Title/Abstract])) OR (trial[Title/Abstract])) OR (randomized controlled trial[Title/Abstract])) OR (RCT[Title/Abstract])) OR (("Clinical Trials as Topic"[Mesh]) OR ( "Clinical Trial" [Publication Type] OR "Controlled Clinical Trial" [Publication Type] OR ("Randomized Controlled Trial" [Publication Type]) OR "Controlled Clinical Trial" [Publication Type] )))))))) OR ("Randomized Controlled Trials as Topic"[Mesh])) AND ((("Sumac"[Title/Abstract]) OR ("Rhus"[Title/Abstract]))) |
| **Scopus**: ( ( TITLE-ABS-KEY ( "Sumuc" ) OR TITLE-ABS-KEY ( rhus ) ) ) AND ( ( ( ( ( TITLE-ABS-KEY ( randomized ) OR TITLE-ABS-KEY ( placebo ) OR TITLE-ABS-KEY ( clinical AND trials ) OR TITLE-ABS-KEY ( randomly ) OR TITLE-ABS-KEY ( trial ) OR TITLE-ABS-KEY ( randomized AND controlled AND trial ) OR TITLE-ABS-KEY ( rct ) ) ) ) ) ) |
| **Web of Science**:  Query #1  **(TS=("rhus")) OR TS=(Sumuc)**  Query #2  **((((((TS=("randomized")) OR TS=("placebo")) OR TS=("clinical trials")) OR TS=("randomly")) OR TS=("trial")) OR TS=("randomized controlled trial")) OR TS=("RCT")**  Query #2  #1 AND #2 |
| **CENTRAL:**  **ID Search Hits**  **#1 (sumac):ti,ab,kw OR (Rhus):ti,ab,kw**  **#2 ("randomized"):ti,ab,kw OR ("placebo"):ti,ab,kw OR ("clinical trials"):ti,ab,kw OR ("randomized controlled trial"):ti,ab,kw OR ("RCT"):ti,ab,kw**  **#3 #1 AND #2 in Trials** |
| **Search strategy date:** February 2025 |
